# Structural-vascular-functional correlation in type 2 non-proliferative macular telangiectasia

**DOI:** 10.1186/s40942-022-00410-3

**Published:** 2022-08-26

**Authors:** Ramesh Venkatesh, Nikitha Gurram Reddy, Pranjal Mishra, Naresh Kumar Yadav, Jay Chhablani

**Affiliations:** 1 Narayana Nethralaya, #121/C, 1st R Block, Chord Road, Rajaji Nagar, Bengaluru, 560010 Karnataka India; 2 University of Pittsburgh School of Medicine, Medical Retina and Vitreoretinal Surgery, 203 Lothrop Street, Suite 800, Pittsburg, PA 15213 USA; 3 Deptartment of Retina and Vitreous, Narayana Nethralaya, #121/C, 1st R Block, Chord Road, Rajaji Nagar, Bengaluru, 560010 Karnataka India

**Keywords:** Macular telangiectasia, Optical coherence tomography angiography, Disease stages, Non-proliferative stage, Visual outcomes

## Abstract

**Purpose:**

To correlate the structural-vascular-functional changes in type 2 non-proliferative macular telangiectasia (MacTel) using optical coherence tomography (OCT) angiography (OCTA).

**Methods:**

In this retrospective study, OCTA and enface OCT image analysis of eyes with confirmed diagnosis of non-proliferative type 2 MacTel was performed. The ‘MacTel area’ was calculated by marking the outer boundary of an area affected by MacTel on superficial (SCP) and deep capillary plexus (DCP) on OCTA images and photoreceptor layer (PRL) on enface OCT scan. At every follow-up OCTA scan visit, best-corrected visual acuity, MacTel area and stage of disease was documented. Analyses between disease stage, MacTel area and logMAR visual acuity was carried out.

**Results:**

In total, 38 single-visit OCTA scans of 22 patients were included. The mean age was 58.9 ± 10.98 years. An increase in disease severity stage correlated positively with MacTel area in SCP segmentations slab (r = 0.334; p = 0.04) and logMAR visual acuity (r = 0.338; p = 0.038). No correlation in the DCP area or PRL area (p > 0.05) was noted with disease stage. A statistically significant positive correlation was noted between the structural changes in PRL layer with vascular changes in SCP (p = 0.021) but not in DCP (p = 0.199). No correlation of visual acuity with changes in SCP, DCP or PRL was noted (p > 0.05).

**Conclusion:**

OCTA is a useful adjunct for determining disease severity in type 2 non-proliferative MacTel by assessing the structural-vascular changes. Further longitudinal studies need to be considered in future for understanding the pathomechanism of retinal damage in type 2 MacTel.

## Introduction

A bilateral perifoveal retinal neurodegenerative and vascular disease that affects the deep capillary vascular network is how macular telangiectasia (MacTel) is most commonly described. It typically appears during the fifth and seventh decades of life, generally in both eyes. It causes abnormalities in both inner and outer retinal components finally resulting in photoreceptor layer atrophy, the growth of retinal pigment clumps (RPC), and aberrant neovascular complexes. [1] The clinical characteristics of type 2 MacTel were identified by Gass and Blodi, who classified them into five stages, starting with loss of perifoveal retinal transparency as stage 1, no/occult visible telangiectasias as stage 2, dilated right angled venules as stage 3, retinal pigment clumps (RPC) as stage 4, and development of subretinal neovascular membrane (SRNVM) as stage 5 [2]. According to Yannuzzi et al., stages 1–4 are non-proliferative and stage 5 is a proliferative stage [3].

OCT and OCT angiography (OCTA) have recently been investigated as promising imaging methods to aid in the diagnosis and characterization of type 2 MacTel [4–9]. Alterations in the photoreceptor layer (PRL) and retinal cavitations have been linked to poor visual acuity. The severity and course of PRL loss is most frequently linked with vision loss in type 2 MacTel [10–12]. Retinal cavitations were recently assessed by Cai et al. using OCT, and they revealed a negative correlation with visual acuity [13]. OCT has been the main tool used to assess PRL involvement [14, 15]. Due to poor outer retinal segmentation and the presence of SRNVM, quantitative PRL assessment in type 2 MacTel is frequently difficult. New prospects for quantitative PRL assessment have been made possible by recent software advancements in ellipsoid zone (EZ) mapping [16].

By examining the motion of flowing red blood cells and thereby allowing the visualization and quantification of functional vessel networks within microcirculatory tissue beds in a non-invasive manner without the use of dye injection, OCTA, a retinal vascular imaging modality, provides in vivo enface microvascular information [17, 18]. Type 2 MacTel has recently been studied using OCTA. The superficial and deep capillary plexuses have been found to be altered in several studies utilizing OCTA to examine the characteristics of type 2 MacTel, including capillary dilatation, aberrant anastomoses, telangiectasias, and decreased capillary density [8, 19, 20]. In non-proliferative type 2A MacTel patients, Saoji et al. attempted to relate the existence of the aberrant vascular network to visual acuity and treatment with intravitreal antivegf injections. They showed that improved response to antivegf injections was associated with the discovery of the aberrant vascular net in the outer retinal segmentation slab on OCTA. Nevertheless, the aberrant vascular changes in various OCTA segmentation slabs of eyes with non-proliferative type 2 MacTel disease with visual acuity were not examined in the study.

In general, the pathophysiology of type 2 MacTel has been described as involving both neurodegenerative and vascular processes. The relationship between the anatomical and vascular alterations in MacTel and functional vision is still unclear. Therefore, it would be helpful to evaluate the vascular alterations in the superficial (SCP) and deep retinal capillary plexuses (DCP) on enface OCTA, correlating with the amount of structural changes in PRL on enface OCT and visual acuity at different disease stages. To the best of our knowledge, there haven't been any research that analyze this data in type 2 MacTel in the literature.

Thus, in this study, we intend to measure the areas of vascular involvement on OCTA, PRL involvement on enface OCT and correlate them with visual acuity in non-proliferative type 2 MacTel using an open-source imaging software Image J.

## Materials and methods

In this retrospective observational study, we reviewed the clinical records and OCTA images of patients diagnosed with type 2 non-proliferative MacTel attending the retina services at a tertiary eye hospital between June 2017 and May 2021. The study complied with the tenets of the Declaration of Helsinki and was approved by the local Institutional Review Board/Ethics Committee. Because the study was a retrospective image analysis, waiver for informed consent was obtained.

Type 2 non-proliferative MacTel was a classification type proposed by Yannuzzi et al. [3] based on clinical features described by Gass and Blodi [2]. Type 2 MacTel was confirmed with other imaging techniques such as confocal blue reflectance and FA obtained using the Spectralis machine (Spectralis, Heidelberg Engineering, Germany). The confirmation of the diagnosis and the decision to include in the study was made by one of the study co-authors (RV). Patients with other concomitant macular pathologies or those with doubtful diagnosis or proliferative type of type 2 MacTel were excluded from the study. Only patients having good quality OCTA images in confirmed cases of non-proliferative type 2 MacTel were included for analysis. The clinical details of each patient were collected retrospectively by review of records, including age, gender, laterality and systemic disease if any. For every eye with OCTA scan, disease stage, best-corrected visual acuity and area of involvement by MacTel on OCTA images in different segmentation slabs viz., SCP, DCP and PRL was documented and calculated.

The Avanti spectral domain OCTA (RTVue-XR Avanti; Optovue, Fremont, CA, USA) was used for retinal micro vessel imaging. Specifically, the signal for kinetic retinal blood was obtained using the SSADA algorithm, an amplitude-based OCT angiography method, which provided decorrelation values for each the vessel so that we could quantitatively evaluate the retinal vasculature [21]. The entire enface microvasculature was evaluated in the 3 × 3 mm area of the parafoveal region and 1 × 1 mm grid at the fovea. Only scans with good signal strength (quality index > 70) were used for analysis. The OCTA and enface OCT images of SCP, DCP and PRL obtained by automated segmentation for SCP and DCP and by careful manual segmentation of PRL were saved in the .jpg/.jpeg format and then exported for further analysis with Image J. The PRL was marked by selecting the upper border at a level just below the external limiting membrane and the lower border at a level of the inner portion of the retinal pigment epithelium (RPE) layer encompassing both the ellipsoid and interdigitation zones [4]. The extent of involvement by type 2 MacTel was noted by identifying abnormal vessel findings in each of the segmentation slab obtained in an automated manner on OCTA. The following features were noted: (1) dilated bulbs on perifoveal vessels, (2) dilatation of perifoveal vessels, (3) vessels distorting foveal avascular zone (FAZ), (4) bunching of vessels, and (5) vascular network in outer retina. These features were previously described in a study by Saoji et al. [19].

## Measurements on Image J

**Fig. 1:**
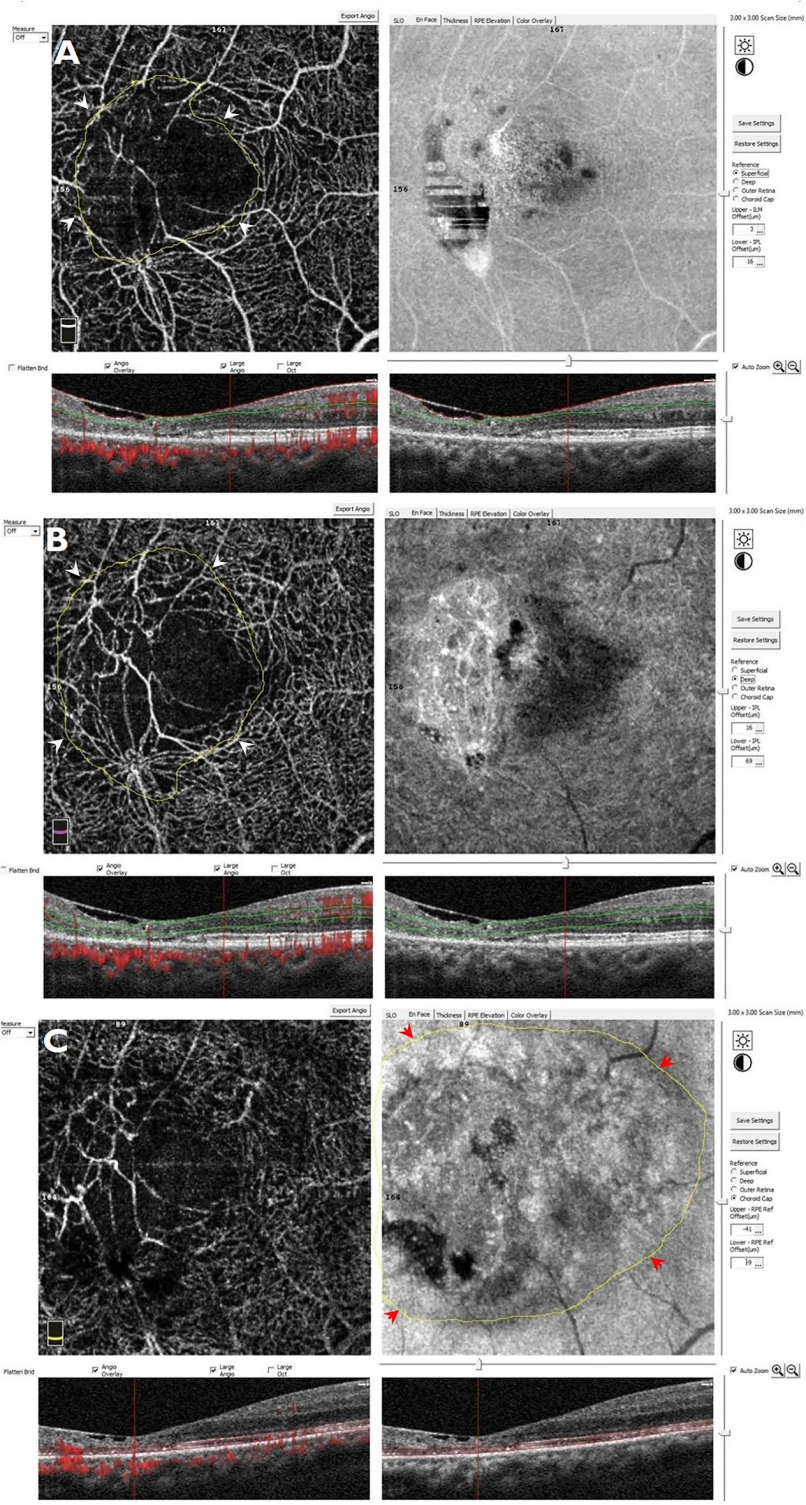
Marking the area of type 2 macular telangiectasia involvement on the optical coherence tomography angiography (OCTA) and enface OCT images. On the OCTA image segmenting through the superficial and deep capillary vascular plexuses, the area of involvement by the type 2 MacTel is identified. The outer boundary of the MacTel area is marked manually (yellow line) using the area tool on the Image J software. The area is measured in sq. millimeter. These are marked by the white arrow heads in the superficial (**A**) and deep capillary plexus slabs (**B**) and by the red arrow heads in the photoreceptor layer slab (**C**)

**Fig. 2:**
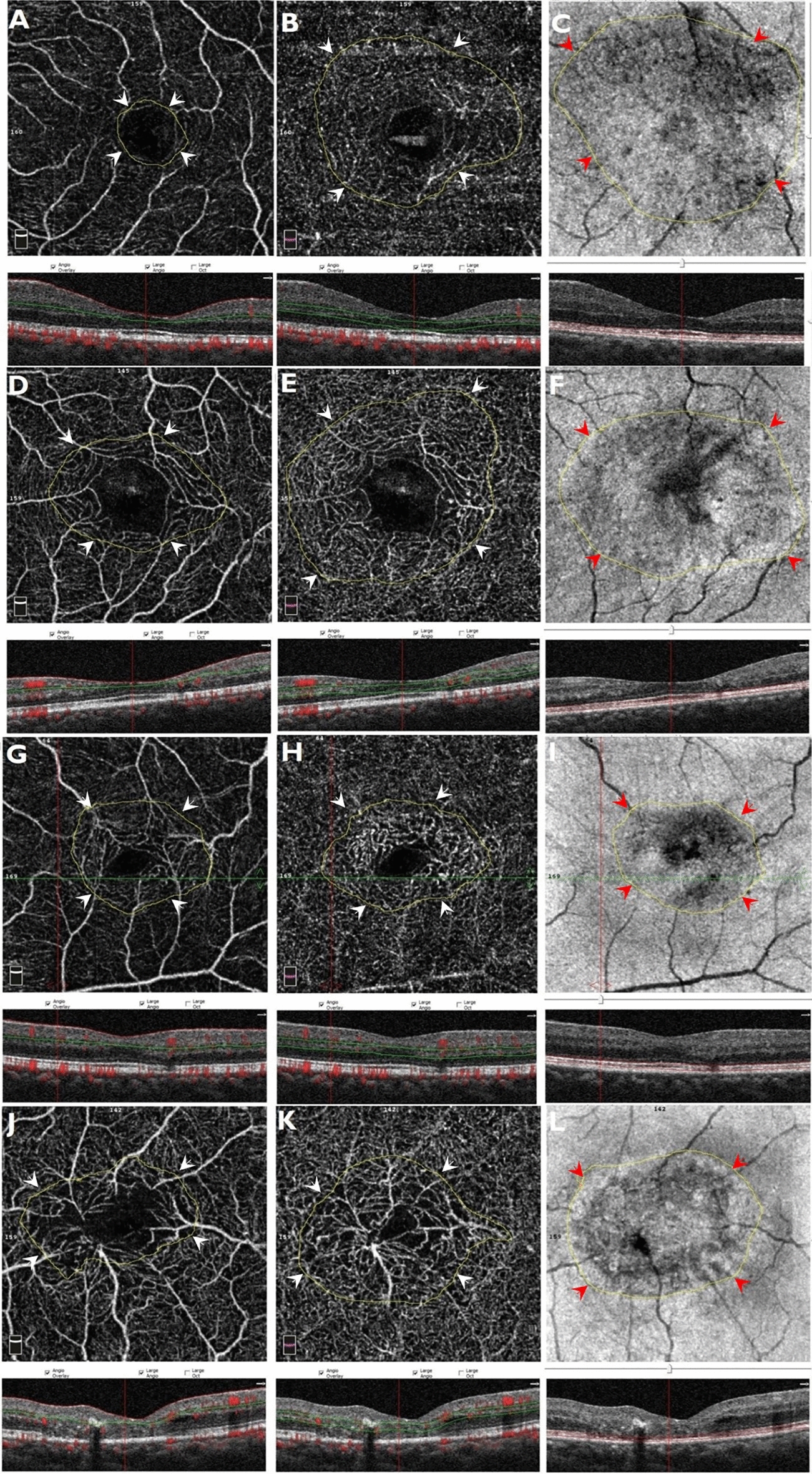
Marking the macular telangiectasia (MacTel) area on the optical coherence tomography angiography (OCTA) and enface OCT images in different non-proliferative disease stages. **A**–**C** MacTel area measurements in disease stage 1. **D**–**F** MacTel area measurements in disease stage 2. **G**–**I** MacTel area measurements in disease stage 3. **J**–**L** MacTel area measurements in disease stage 4. These are marked by the white arrow heads in the superficial and deep capillary plexus slabs and by the red arrow heads in the photoreceptor layer slab

The images were analysed by ImageJ (Open-source software—version 1.53e). On the image J software, all the images were of the same pixels [685 pixels in width X 246 pixels in height]. A scale of 60 pixels per centimeter and pixel aspect ratio of 1.0 was set. Using the polygonal tool, a region of interest (ROI) (i.e., the region involved by type 2 MacTel) was marked and set by the ROI manager in the OCTA image for each segmentation slab (i.e., SCP and DCP separately). Then, the total area of the ROI in square millimeters was calculated using the measure tool. Similarly, the extent of involvement of the PRL damage was marked on the enface OCT image segmenting through the PRL (Figs. 1, 2).

In order to test the repeatability and reproducibility of the measurements on OCTA and enface OCT images, all the measurements in the study were computed by 2 experienced independent graders (RV and NR). The image measurements for the area of involvement by the disease on SCP, DCP and PRL segmentation slabs between the examiners were compared and analysed using the Bland Altman plots. Thereafter, for further analysis, an average of the readings between the two readers was calculated. Analysis between the MacTel area involved on OCTA images, disease stage and visual acuity was planned.

## Statistical tests

All data were analysed using GraphPad Prism version 9.2.0 (332) for Windows, GraphPad Software, San Diego, California USA, www.graphpad.com. The Shapiro–Wilk normality test was used to test the normality of the data sets. Snellen’s vision data was converted to logMAR vision for statistical analysis. Continuous variables like age, logMAR visual acuity and area measurements were described in mean and standard deviation while categorical variables like gender and laterality were described in number and percentage. Non-parametric test like the Kruskal–Wallis test was used to compare the visual acuity and area of involvement by type 2 MacTel between the 4 disease stages. Correlation between the area measurements in different segmentation slabs, disease stage and logMAR visual acuity were analysed using the non-parametric Spearman’s correlation test. P values < 0.05 were considered statistically significant.

## Results

In this study, gradable quality single visit OCTA images were obtained from 38 eyes of 22 patients diagnosed with type 2 non-proliferative MacTel. There were 3 eyes showing proliferative disease stage and 1 eye with poor quality OCTA scans due to cataract. These eyes had to be excluded from the study analysis. There was an equal distribution of males and females in the study. The mean age of presentation of patients was 58.9 ± 10.98 years. 63% of the patients were diabetics. The number of right eye and left eye OCTA images in the study was 22 and 16 respectively. The mean logMAR visual acuity was 0.23 ± 0.26 (Snellen Equivalent = 20/34) (Table 1).

**Table 1:** Demographic data

No. of patients with non-proliferative type 2 MacTel	22
Males:Females	11:11
Mean age (years)	58.9 ± 10.98
Number of eyes with OCTA scans	38
Laterality (Right:Left)	22:16
No. of patients with diabetes mellitus	14(63%)
Mean logMAR visual acuity	0.23 ± 0.26
Snellen equivalent	20/34

*OCTA* optical coherence tomography angiography

**Fig. 3:**
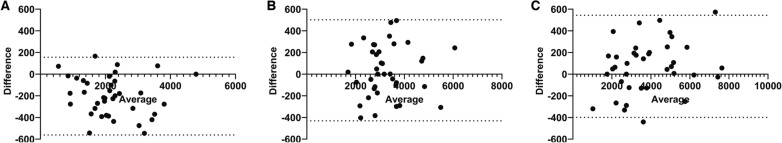
Bland Altman (Difference vs Average) plots. Bland–Altman Plot between reader 1 and 2 for **A** superficial capillary plexus measurements; **B** deep capillary plexus measurements and **C** photoreceptor layer measurements

The Bland Altman plots showed low average bias between the readers for the measurements (Table 2 and Fig. 3). There were 8 eyes showing disease stage 1, 3 eyes with disease stage 2, 19 eyes with stage 3 and 8 eyes showing stage 4 disease.

**Table 2:** Bland Altman comparison between 2 readers for area measurements on Image J

	SCP	DCP	PRL
Bias (microns)	− 202.8	35.44	72.18
SD of Bias	183.1	238.0	240.6
95% limits of agreement			
From	− 561.6	− 431.0	− 399.3
To	156.1	501.9	543.7

*SCP* superficial capillary plexus, *DCP* deep capillary plexus, *PRL* photoreceptor layer, *SD* standard deviation

Table 3 describes the differences in visual acuity and area measurement between the different disease stages. Statistically significant difference was noted in visual acuity between the four disease stages (p = 0.045). In SCP and DCP segmentation slabs, the area of involvement measured was more in the early stages of the disease (stages 1–3) while it reduced in the more advanced stages (stage 4). However, these differences were not statistically significant (SCP; p = 0.161 and DCP; p = 0.477). A larger area of involvement in PRL slab was noted in stage 3 of the disease. Significant positive correlation was noted between the disease stage and logMAR visual acuity (r = 0.338; p = 0.038) and SCP area involvement (r = 0.334; p = 0.04). The DCP area (p = 0.128) and PRL area (p = 0.648) measurements showed negative correlation with advancing disease stage but they were not statistically significant (Table 4). Table 5 demonstrate the relationship between the SCP and DCP area involvement on OCTA with the extent of affected PRL on enface OCT images. Significant positive correlation was noted for SCP area measurements (r = 0.372, p = 0.021) with the extent of affected PRL. The DCP area measurements showed positive correlation with affected PRL area but the measurements were not statistically significant (r = 0.213, p = 0.199). In Table 6, we note the correlation between visual acuity and area involvement across different disease stages in SCP, DCP and PRL. No statistically significant correlation was noted between these parameters.

**Table 3:** Disease stage wise changes in visual acuity and area involved by type 2 MacTel using Spearman’s correlation test

	Stage 1	Stage 2	Stage 3	Stage 4	P value
No. of visits	8	3	19	8	
logMAR VA	0.108 ± 0.208	0.00	0.286 ± 0.233	0.299 ± 0.337	0.045
SCP area (mm^2^)	1900 ± 838.3	1512 ± 835.7	2142 ± 824.3	2756 ± 1019	0.161
DCP area (mm^2^)	3547 ± 828.4	3946 ± 1393	3175 ± 1069	2882 ± 445	0.477
PRL area (mm^2^)	3183 ± 1551	3557 ± 344	4682 ± 1753	2693 ± 637.3	0.014

*SCP* superficial capillary plexus, *DCP* deep capillary plexus, *PRL* photoreceptor layer, *VA* visual acuity

**Table 4:** Correlation of disease stage with logMAR visual acuity and area involved using Spearman’s correlation test

Stage vs	LogMAR VA	SCP area	DCP area	PRL area
r	0.338	0.334	− 0.251	− 0.0766
95% confidence interval	0.011 to 0.600	0.007 to 0.597	− 0.536 to 0.084	− 0.395 to 0.258
P value	0.038	0.04	0.128	0.648

*SCP* superficial capillary plexus, *DCP* deep capillary plexus, *PRL* photoreceptor layer, *VA* visual acuity

**Table 5:** Correlation of superficial and deep capillary area involvement on OCT angiography with the extent of photoreceptor layer loss on enface OCT images across all stages

PRL vs	SCP	DCP
r	0.372	0.213
95% confidence interval	0.05 to 0.624	− 0.124 to 0.506
P value	0.021	0.199

*SCP* superficial capillary plexus, *DCP* deep capillary plexus, *PRL* photoreceptor layer

**Table 6:** Correlation of logMAR visual acuity with superficial and deep capillary plexus area involvement on OCT angiography and involvement of photoreceptor layer damage on enface OCT

logMAR VA vs	SCP	DCP	PRL
r	0.178	0.099	0.076
95% confidence interval	− 0.160 to 0.478	− 0.237 to 0.414	− 0.259 to 0.395
P value	0.285	0.554	0.65

*SCP* superficial capillary plexus, *DCP* deep capillary plexus, *PRL* photoreceptor layer, *VA* visual acuity

## Discussion

The results of this study suggest that with advancing stage of non-proliferative type 2 MacTel, there is worsening in visual acuity and increased involvement of SCP. The area of PRL involvement did not correlate with the disease stage, visual acuity and extent of DCP involvement in the current study; however, it showed a significant positive correlation with SCP involvement.

Type 2 MacTel is primarily a disease of the DCP. The pathophysiology of type 2 MacTel is often linked to Müller cell death or dysfunction, which later leads to perifoveal foveal capillary proliferation, photoreceptor damage and finally PRL disruption [1, 22]. The PRL integrity has been correlated with visual acuity and prognosis across a wide variety of retinal and choroidal diseases [23–26]. Even in type 2 MacTel, PRL discontinuity has shown to be associated to poor functional outcomes [4, 15]. However, studies have examined PRL discontinuity as a binary measure as to whether it is intact or disrupted. Quantification of the PRL discontinuity has not been routinely described and is not currently available easily. A small number of studies have reported the area of PRL involvement on the enface scans and another study has measured the EZ area and volume involved using the EZ mapping software [5, 6, 9]. To the best of our knowledge, we did not find any literature which correlated the PRL area involvement with vascular plexus involvement in SCP and DCP in different disease stages of type 2 MacTel. The uniqueness of this study is the measurement of damage due to MacTel on the enface images which provides a complete picture of the disease involvement at different retinal layers. Our study adds to the current knowledge of PRL disruption in type 2 MacTel by measuring the area of involvement from exported images using an open-source imaging software Image J and then correlating it with vascular involvement, visual acuity and disease stage.

In this study, we noted the extent of PRL involvement in eyes with non-proliferative type 2 MacTel did not show correlation stage of disease. Across stages 1–3, there was a progressive increase in the area of involvement of PRL damage. In stage 4 disease characterized by the presence of retinal pigment clumps, we noted a decrease in area of PRL involvement. This could be plausibly explained as follows: With advancing disease stages, there is PRL atrophy which causes RPE cell migration towards its center. When the RPE cells come in contact with these abnormal telangiectatic vessels, the cells use them as a scaffold and migrate along the vessels towards the inner retina, where they proliferate and spread horizontally away from the vessels along the retinal layers, usually at the level of the outer plexiform layer. The pigment cells grow sideways faster than vertically [27, 28]. Thus, the horizontal spread is larger; thereby, casting a larger shadow on the underlying photoreceptor layer and masking the true extent of PRL damage on the enface OCT scans. Considering this, we measured only the extent of visible PRL damage and excluded the area occupied by projection artefacts due to RPC which was a part of the area with PRL damage.

We also noted that the PRL area on enface OCT correlated positively with the SCP area in the study. This was primarily noted across disease stages 1–3. With progression in the disease severity stage from 1 to 3, there was increased involvement of SCP and PRL layers. This suggests that the type 2 MacTel disease which is primarily affects the DCP progresses further towards the superficial retina by affecting the SCP and outer retina by affecting the photoreceptor layer.

On studying the visual acuity correlation with area of involvement in the different segmentation slabs, we did not find any statistically significant correlation between the two variables. Type 2 MacTel is a perifoveal disease routinely sparing the foveal photoreceptor and retinal pigment epithelium layers, especially in the early non-proliferative stages. Also, we measured the enface area across the different retinal segmentation slabs and not the cross-sectional area across the retinal layers. As per MacTel Project report no. 8, the reasons for severe vision loss (< 20/200) in both varieties of type 2 MacTel were described. They analysed the cross-sectional images such as colour fundus photographs, fluorescein angiography images and OCT scans and identified larger extent of PRL atrophy, larger area of MacTel involvement, presence of proliferative disease and presence of retinal pigment clumps and macular hole as the most important factors for poor vision in type 2 MacTel [10]. In our study, we limited our inclusion criteria only with non-proliferative disease stage. Hence, we found no relationship between visual acuity and area of involvement in different retinal layers in the study.

Using recent software developments such as EZ mapping, Runkle et al. studied the EZ-RPE thickness, EZ-RPE central foveal area and EZ-RPE central subfield volume on OCTA and correlated these parameters with visual acuity. They concluded that abnormalities in EZ-RPE thickness, area, and volume affected the visual acuity in type 2 MacTel [9]. In our study, we measured the area of PRL involvement using simple and easily available open-source software such as Image J. Not only did we measure PRL disruption area but we also measured the area of vascular involvement in SCP and DCP and correlated between them. No correlation was noted between the visual acuity and areas of involvement in SCP, DCP and PRL in our study. Thus, our study brings out the point that in type 2 MacTel, the structural damage and vascular loss are not related to each other and have no bearing on the visual acuity, especially in non-proliferative eyes.

There are a few important limitations to this study. The sample size of patients with type 2 MacTel enrolled with good quality OCTA scans is small. In addition, the image data which we present here represents a cross-sectional view of the disease. We did not have data showing the longitudinal follow-up of these patients. Therefore, it was not possible to determine how parameters such as PRL measurements or capillary vessel density would change over the disease course. We did not separately measure the foveal PRL loss on the enface OCT scans and correlate them with the vascular changes seen in the SCP and DCP or with visual acuity. We found it extremely challenging to accurately locate the fovea on the enface OCT scans in the presence of significant surrounding perifoveal PRL loss. Also, using only subjective Snellen visual acuity for testing the functional outcome is not the ideal parameter. Using additional functional testing techniques such as microperimetry or multifocal electroretinogram may provide a more sensitive measurement of retinal and visual function in type 2 MacTel [11, 29]. Despite these limitations, we believe that this study provides a novel contribution in the diagnosis and understanding of type 2 non-proliferative MacTel disease by assessing its structural–functional-vascular relationship using a non-invasive imaging modality.

To conclude, OCTA is a useful adjunct for determining disease severity in type 2 non-proliferative MacTel by assessing the structural-vascular-functional relationship. Further longitudinal studies with larger number of cases needs to be considered in future for understanding the pathomechanism of retinal damage in type 2 MacTel.

## Data Availability

The datasets used and/or analysed during the current study are available from the corresponding author on reasonable request.

## Abbreviations

MacTel: Macular telangiectasia
OCT: Optical coherence tomography
OCTA: Optical coherence tomography angiography
RPC: Retinal pigment clumps
RPE: Retinal pigment epithelium
PRL: Photoreceptor layer
EZ: Ellipsoid zone
SCP: Superficial capillary plexus
DCP: Deep capillary plexus

## Declarations

### Animal research

This article does not contain any studies with animals performed by any of the authors.
